# Risk Score Elaboration for Stroke in Cardiac Surgery

**DOI:** 10.21470/1678-9741-2020-0331

**Published:** 2021

**Authors:** Ellen Hettwer Magedanz, João Carlos Vieira da Costa Guaragna, Luciano Cabral Albuquerque, Mario Bernardes Wagner, Fernanda Lourega Chieza, Natalia Lamas Bueno, Luiz Carlos Bodanese

**Affiliations:** 1 Cardiology Service, Faculdade de Medicina, Pontifícia Universidade Católica do Rio Grande do Sul (PUCRS), Porto Alegre, RS, Brazil.

**Keywords:** Stroke, Risk Factors, Cardiac Surgical Procedures, Postoperative Period, Arterial Occlusive Diseases

## Abstract

**Introduction:**

Stroke is a complication that causes considerable morbidity and mortality during the heart surgery postoperative period (incidence: 1.3 to 5%; mortality: 13 to 41%). Models for assessing the risk of stroke after heart surgery have been proposed, but most of them do not evaluate postoperative morbidity. The aim of this study was to develop a risk score for postoperative stroke in patients who undergo heart surgery with cardiopulmonary bypass.

**Methods:**

A cohort study was conducted with data from 4,862 patients who underwent surgery from 1996 to 2016. Logistic regression was used to assess relationships between risk factors and stroke. Data from 3,258 patients were used to construct the model. The model’s performance was then validated using data from the remainder of the patients (n=1,604). The model’s accuracy was tested using the area under the receiver operating characteristic (ROC) curve.

**Results:**

The prevalence of stroke during the postoperative period was 3% (n=149); 59% of the patients who exhibited this outcome were male, 51% were aged ≥ 66 years, and 31.5% of the patients died. The variables that remained as independent predictors of the outcome after multivariate analysis were advanced age, urgent/emergency surgery, peripheral arterial occlusive disease, history of cerebrovascular disease, and cardiopulmonary bypass time ≥ 110 minutes. The area under the ROC curve was 0.71 (95% confidence interval 0.66 - 0.75).

**Conclusion:**

We were able to develop a risk score for stroke after heart surgery. This score classifies patients as low, medium, high, or very high risk of a surgery-related stroke.

**Table:** 

Abbreviations, acronyms & symbols	
**AF** **CABG** **CI** **COPD** **CPB** **CVD** **DM** **HL** **IL** **NNE** **OR** **PACK2** **PAOD** **ROC** **SD**	**= Atrial fibrillation** **= Coronary artery bypass grafting** **= Confidence interval** **= Chronic obstructive pulmonary disease** **= Cardiopulmonary bypass** **= Cerebrovascular disease** **= Diabetes mellitus** **= Hosmer-Lemeshow chi-square goodness-of-fit test** **= Interleukin** **= Northern New England** **= Odds ratio** **= Priority, arteriopathy, cardiac, kidney** **= Peripheral arterial occlusive disease** **= Receiver operating characteristic** **= Standard deviation**

## INTRODUCTION

The profile of heart surgery patients is going through a process of progressive change; the age of this population is increasing, their clinical conditions are becoming more severe, and they present with a wide range of associated comorbidities, making them an increasingly complex group of patients ^[1]^. In conjunction with this situation, cardiology, both clinical and interventionist, is advancing.

However, despite technological developments and improvements in heart surgery techniques, complications such as cerebral vascular accident (stroke) remain a challenge, causing high rates of morbidity and mortality. Stroke is a heart surgery complication responsible for considerable mortality and morbidity, with estimated incidence rates in the literature from 1.3 to 4.3% and mortality rates in the range of 13 to 41% ^[1-3]^. It extends the duration of intensive care and increases the length of in-hospital stay and the need for home care, raises hospital costs, and has a major impact on the quality of life of survivors ^[1]^.

In both the domestic Brazilian literature and the international literature, advanced age is a predictor of risk that is robustly associated with stroke in heart surgery and the risk of stroke among octogenarian patients can be as high as 9%. It is believed that the increase in age is proportional to the increase in comorbidities that predispose to atherosclerosis, which, in turn, increases the risk of a perioperative neurological event ^[4]^.

Other conditions have also been identified as important risk factors for stroke, such as history of cerebrovascular disease (CVD), carotid disease, peripheral vascular disease, systemic arterial hypertension, diabetes mellitus (DM), atrial fibrillation (AF), urgent/emergency surgery, and increased duration of cardiopulmonary bypass (CPB) ^[1-6]^.

Measurement and monitoring of immediate results after heart surgery are essential to measure the efficacy of procedures and to determine whether results are in line with established quality programs ^[7]^. Although more than 100 studies have been conducted for risk stratification and/or perioperative prognosis, such as the European System for Cardiac Operative Risk Evaluation (or EuroSCORE) ^[8]^, for example, few models also cover postoperative morbidity.

The objective of this study was to develop a model for a risk score for postoperative stroke among adult patients who undergo heart surgery with CPB at a University Hospital in the South of Brazil.

## METHODS

We conducted a historical cohort observational study based on variables obtained from the postoperative ward’s database of cardiac surgery at the Hospital São Lucas - Pontifícia Universidade Católica do Rio Grande do Sul (or PUCRS), according to the principles established in the Declaration of Helsinki and approved by the Research Ethics Committee under No. 12403413.0.0000.5336. We included 4,862 patients who underwent heart surgery with CPB between January 1996 and December 2016. Patients who underwent congenital heart surgery were excluded.

The variables initially tested in the statistical analyses were: age, sex (male and female), urgent/emergency surgery (included as a single variable and defined as a need for intervention within 48 hours); peripheral arterial occlusive disease (PAOD); surgery type (coronary artery bypass grafting [CABG] or valve replacement); AF; history of CVD, defined as a patient history of stroke, transient ischemic attack, or surgical repair (carotid endarterectomy), ≥ 50% luminal stenosis of the carotid artery seen on angiography, echography, or magnetic resonance angiography, or any combination of these; DM; chronic obstructive pulmonary disease (COPD), diagnosed clinically by chest X-ray and/or spirometry and/or on the basis of drug treatment (corticoid or bronchodilator); obesity (body mass index ≥30 kg/m^2^); hypertension; surgical reintervention; and CPB time (classified as ≥110 minutes). Initial analysis of the variables followed a hierarchical model based on biological plausibility and the results of studies published previously ^[1,4,6,7]^ indicating the relevance and strength of associations between these potential risk factors and the occurrence of the outcome being studied (intrahospital stroke).

The main outcome was patients with Type I stroke during the immediate postoperative period and up to 30 days. Type I neurological deficit (stroke) was classified at our service as any new neurological deficit > 24 hours, confirmed by a clinical examination conducted by a neurologist and a cerebral imaging exam (computed tomography or magnetic resonance imaging), or stupor or coma at the time of discharge, based on the classification of the American College of Cardiology (or ACC)/American Heart Association (or AHA) guidelines ^[9]^.

### Statistical Analysis

Continuous data were described by mean ± standard deviation. Categorical variables were presented as counts and percentages. Univariate comparisons were made with *t*-tests, chi-square tests, or Fisher’s exact tests, as appropriate. Data were randomly split into a development dataset (2/3) and a validation dataset (1/3). Potential explanatory variables were selected based on the literature and clinical grounds or on a hypothesis about their relationship with prolonged mechanical ventilation. A multiple logistic regression with backward selection was fitted to the development dataset to identify independent risk factors for prolonged mechanical ventilation. Candidate variables with a *P*-value < 0.10 were entered into this development model.

The development model was then tested on the validation dataset using three statistical procedures: C-statistic (area under the receiver operating characteristic [ROC]) curve, Hosmer-Lemeshow chi-square goodness-of-fit test (HL), and Pearson’s correlation coefficient between the events observed and those predicted by the model.

After observing a successful validation exercise, the development and validation datasets were combined. In this process, variables were not included or removed, resulting in more precise values for previously estimated coefficients. A weighted risk score was created from this final model by rounding the adjusted odds ratios (OR) to their nearest integer. These values were then summed. In all cases, a *P*-value < 0,05 was deemed statistically significant. Statistical analyses were performed using IBM Corp. Released 2013, IBM SPSS Statistics for Windows, Version 22.0, Armonk, NY: IBM Corp. and R for Windows, version 3.4.2 (R Development Core Team, www.r-project.org).

## RESULTS

### Characteristics

Three percent (n=149) of the entire sample of patients (n=4,862) suffered a stroke during the postoperative period. Of these patients who exhibited the outcome, 59.1% were male, 51% were aged ≥ 66 years, and 31.5% died. The mean age of the study population was 58.9±12 years. The variables urgent/emergency surgery, PAOD, AF, history of CVD, DM, hypertension, COPD, surgical reintervention, CPB time, and death all exhibited statistical significance in univariate analysis. Around three-quarters of the sample were patients who had undergone CABG without valve replacement. Table 1 shows the results of the univariate analyses for patient characteristics and risk of stroke.

**Table 1:** Univariate analysis of study groups (n=4,862).

Variables	Stroke (%)	No stroke (%)	*P* -value
Surgery type			
CABG	109 (73.2)	3398 (72.1)	
Valve replacement	26 (17.4)	1054 (22.4)	0.067
CABG + valve replacement	14 (9.4)	261 (5.5)	
Age			
18-50 years	10 (6.7)	957 (20.3)	
51-65 years	63 (42.3)	2180 (46.3)	< 0.001
≥ 66 years	76 (51.0)	1576 (33.4)	
Age (mean ± SD)	64.2 ± 10.4	59.1 ± 12.6	
Male	88 (59.1)	3007 (63.8)	0.261
Emergency/urgent surgery	20 (13.4)	285 (6.0)	0.001
PAOD	27 (18.1)	264 (7.7)	< 0.001
Atrial fibrillation	19 (12.8)	339 (7.2)	0.016
History of CVD	32 (21.5)	285 (6.0)	< 0.001
Diabetes	51 (34.2)	1154 (24.5)	0.009
Hypertension	109 (73.2)	3059 (64.9)	0.044
COPD	33 (22.1)	708 (15.0)	0.021
Obesity	19 (12.8)	516 (10.9)	0.505
Reintervention	21 (14.1)	275 (5.8)	0.001
CPB time ≥ 110 min.	48 (32.2)	897 (19.2)	< 0.001
Death	47 (31.5)	397 (8.4)	< 0.001

CABG=coronary artery bypass grafting; COPD=chronic obstructive pulmonary disease; CPB=cardiopulmonary bypass; CVD=cerebrovascular disease; PAOD=peripheral arterial occlusive disease; SD=standard deviation

### Development of Preliminary Risk Model

A multiple logistic regression was performed with data from 3,258 non-consecutive patients (selected at random), equating to 2/3 of the whole sample. The predictors selected (according to the selection criteria described in Methods) to construct the score were age, urgent/emergency surgery, PAOD, history of CVD, and CPB time ≥ 110 minutes.

### Validation of the Risk Model

The external validation was conducted with data from 1,604 patients (1/3 of the entire sample) selected at random. The risk model’s accuracy as measured by the area under the ROC curve was 0.74 (95% confidence interval [CI] 0.67 - 0.82) showing that it has good discriminatory power.

### Risk Model Based on the Entire Sample (N=4,862)

Once it had been observed that the preliminary model was performing appropriately in the validation process, the model was recalculated by combining the two databases (preliminary and validation) to obtain the final score. No variables were added or removed as part of this process, which simply produced more precise values for the coefficients previously estimated. A multiple logistic regression was conducted with the variables listed, resulting in a recalibrated risk score (Tables 2 and 3).

**Table 2:** Logistic regression (data for the entire sample, n=4,862).

Variables	OR	95% CI	*P* -value*
Age			
< 51 years	1	-	-
51 to 65 years	2.3	1.2 - 4.6	0.014
≥ 66 years	3.6	1.8 - 6.9	< 0.001
Urgent/emergency surgery	2.1	1.3 - 3.5	0.003
PAOD	1.8	1.1 - 2.8	0.010
History	3.4	2.2 - 5.2	< 0.001
CPB time ≥ 110 min	1.7	1.2 - 2.5	0.002

* *P* -value=statistical significance according to the Wald test (n=4,862, events=149).

CI=confidence interval; CPB=cardiopulmonary bypass; OR=odds ratio; PAOD=peripheral arterial occlusive disease

**Table 3:** Multivariate risk score calculated using the entire sample (n=4,862).

Preoperative characteristics	Point*
Age	
< 51 years	0
51 to 65 years	1
≥ 66 years	3
Urgent/emergency surgery	1
PAOD	1
History of CVD	2
CPB time ≥ 110 min	1

* Obtained by rounding the odds ratios from the logistic model for the entire sample.

CPB=cardiopulmonary bypass; CVD=cerebrovascular disease; PAOD=peripheral arterial occlusive disease

**Fig. 1:**
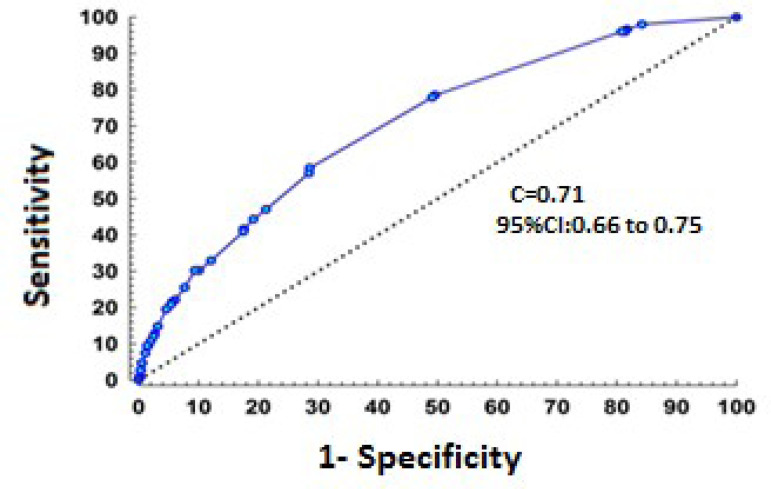
Area under the receiver operating characteristic (ROC) curve for detection of the outcome stroke. C=area under the ROC curve; 95% confidence interval (CI)=0.71 (0.66 - 0.75) for the final risk model (n=4,862).

Factors associated with increased risk of postoperative stroke were age ≥ 66 years (three points), history of CVD (two points), age in the range of 51 to 65 years, urgent/emergency surgery, PAOD, and CPB time ≥ 110 minutes (each scoring one point). The area under the ROC curve for the score was 0.71 (95% CI 0.66 - 0.75) (Figure 1).

**Fig. 2:**
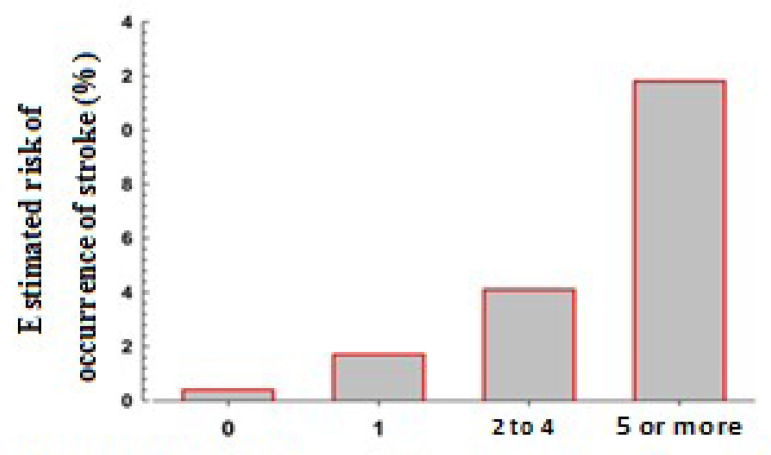
Stroke risk score categories and distribution of risk in 4,862 patients.

Table 4 lists the risk of stroke according to the score and the risk classification (summing scores). In the entire sample, 50.3% of the heart surgery patients had a high risk or very high risk assessment, with probability of suffering a stroke during the postoperative period estimated by the score at 4.1% and 11.8%, respectively. The bar graph (Figure 2) shows the predicted stroke rate according to the risk score classes.

**Table 4:** Risk of stroke according to the score (n=4,862).

Score*	Sample (n=4,862)	Stroke		Risk category
		n	%	
0	756	3	0.4	Low
1	1,659	29	1.7	Medium
2 to 4	2,219	90	4.1	High
5 or more	228	27	11.8	Very high

* The resulting logistic model provides direct estimates of the probability of occurrence of the outcome; data were processed and analyzed with the aid of the IBM Corp. Released 2013, IBM SPSS Statistics for Windows, Version 22.0, Armonk, NY: IBM Corp.

**Fig. 3:**
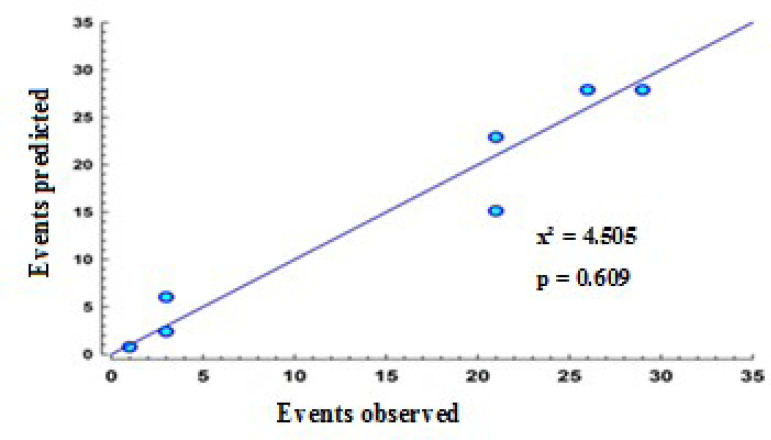
Distribution of points representing the outcome (stroke) as predicted by the logistic model and observed in the patient sample.

In order to test the model’s calibration, the observed rate of stroke was compared with the predicted rate for all patients in each of the score’s four risk classification intervals (Figure 3), resulting in a coefficient for the predicted/observed correlation of 0.98 with x² = 4.505 (*P*=0.609) (HL).

## DISCUSSION

Stroke is a severe and much feared postoperative complication, particularly after heart surgery, and it can have considerable impact on patients’ functional capacity, with economic and social repercussions. This study constructed a risk score for stroke during the postoperative period after heart surgery, starting with a selection of variables described in previous studies ^[2,4,6,10]^, of which the following were identified as predictors of risk: age > 51 years, urgent/emergency surgery, PAOD, history of CVD, and CPB time ≥ 110 minutes.

The prevalence of postoperative stroke observed in this study was 3%, which is similar to rates reported in the literature, varying from 1.4% in single surgery to 14% among patients who undergo CABG together with valve replacement ^[10,11]^. A Spanish multicenter study ^[12]^ of 26,347 patients treated with CABG reported 5% rate of occurrence of perioperative stroke, similar to the findings in our sample, and higher than the rate described by Mérie et al. ^[1]^, who studied 33,062 patients over nine years and observed a 1.6% incidence of stroke ^[12]^. A study with 108,711 patients who had CABG with CPB observed a 1.8% rate of stroke in the first 30 days of the postoperative period ^[14]^. In the present study, mortality among patients who suffered a stroke after heart surgery was 31.5%, which is a higher rate than reported in other studies ^[14]^. Countless factors have been associated with occurrence of stroke after heart surgery ^[2,4,6]^, but there is no consensus in the literature on which are the most important, or to what extent these factors are independent predictors of risk of postoperative CVD.

Many studies have demonstrated that advanced age is an independent risk factor for occurrence of stroke ^[4,6,15,16]^. It is believed that the increasing age of patients currently treated with heart surgery, which is a consequence of increasing longevity, is associated with more comorbidities and greater susceptibility to cerebrovascular complications ^[3]^. Carrascal et al. ^[4]^ conducted a study with the objective of identifying the influence of age on the incidence of stroke after CABG and found a 4.1% rate of the outcome among octogenarian patients, compared to 3.5 % in other patients. Whitlock conducted a study ^[14]^ that found age ≥ 65 years to be an independent predictor of risk of stroke, with an OR of 1.9 (95% CI 1.8 - 2.0) ^[14]^. Age is also a predictive factor of risk of stroke in the majority of risk scores in other studies ^[3,6,8,12]^.

In this study, advanced age was the risk factor with the greatest impact on occurrence of postoperative stroke, adding one point to scores for patients aged 51 to 65 years and three points for patients aged ≥ 66 years. This finding agrees with published data and it is supposed that elderly patients may have increased loss of vascular tone, causing intimal fragility and making atherosclerosis more likely and, as a consequence, increasing the risk of a neurological event ^[4]^.

A history of CVD was also an important risk variable in this study, increasing the score by two points, with an OR of 3.4 (95% CI 2.2 - 5.2). CVD prior to heart surgery has been described in the literature as an important risk factor associated with stroke during the postoperative period ^[14,17,18]^. Whitlock et al. ^[14]^ also identified CVD as an important predictor of risk in a study including more than 100 thousand patients treated with heart surgery, showing it was an independent risk factor for the outcome with an OR of 2.1 (95% CI 1.9 - 2.3). In a study that constructed a score for stratification of stroke risk in patients with AF who underwent CABG, CVD was an important risk variable, scoring two points, similar to the findings of our study ^[19]^. Some authors state that abnormal findings on preoperative imaging exams, such as presence of multiple infarctions, can also contribute to increased risk of stroke during the postoperative period ^[19,20]^.

In our score, surgical priority (urgent/emergency surgery) also had an impact on the proposed risk model and was present in 14.2% of the patients who suffered a stroke during the postoperative period. In a risk score proposed by Northern New England (NNE), based on a population who underwent CABG only, surgical priority had a strong relationship with the outcome - OR 2.49 (95% CI 1.82 - 3.39), *P*<0.001 -, contributing 2.5 points in the risk model. In a model developed by Hornero et al. ^[12]^, urgent/emergency surgery was an independent risk factor for the outcome and, just as in our study, this variable was worth one point in the PACK2 (priority, arteriopathy, cardiac, kidney) score.

Another variable that was included in our score was PAOD, with an OR of 1.8 (95% CI 1.1 - 2.8). The condition added one point to a patient’s score. Thus, as in our study, the risk estimate proposed by another author also included arteriopathy as a risk variable, and it was worth one point in their score. In the NNE score ^[6]^, arteriopathy was part of a variable called vascular disease, which also included CVD and was present in 29% of the patients who suffered a stroke during the postoperative period, and was worth two points on this risk score. In our study, the variables PAOD and CVD were scored separately. It is believed that most of surgical patients have atherosclerotic disease established in different vascular beds, including peripheral vascular disease and carotid disease, even if subclinical, which increases the risk of stroke during the postoperative period. The rate of occurrence of stroke in patients with carotid disease who undergo CABG is 9% and it is considered an important predictor of risk ^[21]^. It is recommended that the possibility of carotid disease should be investigated in preoperative workup of patients with peripheral vascular disease, as a mean of preventing stroke during the postoperative period ^[22]^.

CPB time ≥ 110 minutes was associated with stroke during the postoperative period and 32.2% of the patients who had this variable exhibited the outcome. Other study reported a similar result, showing that 32% of patients who had similar CPB time suffered neurological damage ^[1]^.

Neurological changes related to CPB time have been widely studied and its complications are well-established in clinical practice, where many pathophysiologic changes take place, including coagulation disorders and activation of the inflammatory cascade caused by mechanical stress to the elements involved in changed blood flow, triggering release of cytokines such as tumour necrosis factor alpha, interleukin (IL)-6, and IL-8 ^[23,24]^.

The changes to blood flow and distribution that occur during CPB, concomitant reduction of pump flow rate to facilitate surgical repair, and the patient’s response to these changes can all affect cerebral perfusion, predisposing the patient to cerebrovascular complications ^[23,25]^.

In constructing this risk score, one point was attributed to CPB time ≥ 110 minutes, with an OR of 1.7 (95% CI 1.2 - 2.5). Many studies have suggested a relationship between CPB time and CVD after surgical procedures ^[1,3,25]^. Tarakji et al. ^[22]^ conducted a study with 45,432 patients, demonstrating that use of CPB increased the likelihood of the outcome fivefold. The authors agreed that the relationship between CPB and CVD is caused by inflammatory factors.

Many studies have included variables such as DM, AF, hypertension, surgical reintervention, and COPD in their scores ^[2,6]^. However, while these variables did exhibit statistical significance in the univariate analysis in this study, they were not identified as independent predictors of risk after multivariate analysis and were, therefore, excluded from the final risk model.

According to the ROC curve analysis, the discriminatory power of the model developed in the present study was 0.71 (95% CI 0.66 - 0.75). The score’s calibration, representing the degree of agreement between predicted risk of stroke and observed strokes (HL), was r = 0.98. If the area under the curve is ≥ 0.7, it can be stated that a model has acceptable discriminatory power and can be used to classify patients ^[25]^.

### Limitations

Among the limitations of this study, the analysis is based on a sample of patients from a single institution, which could have an influence on accuracy. The limited number of patients may have affected identification of variables relevant to the analysis, which can be dealt with in future studies. We suggest validating the score in an external population, with data from other institutions, so that it can have broad clinical application.

Notwithstanding, our objective was to develop a score that reflects the situation in our setting and compare it with published data. There is significant scientific interest in the best possible preoperative assessment of heart surgery patients, in order to define risk of stroke during the postoperative period. The results can be used to guide preventative measures to avoid damaging neurological events that compromise patient survival and quality of life.

## CONCLUSION

We used the clinical and surgical variables identified in our study (age, surgical priority, PAOD, history of CVD, and CPB time ≥ 110 minutes) to develop a score that can establish the risk of stroke after heart surgery. The resulting score classifies patients as low, medium, high, or very high surgical risk of the cerebrovascular event stroke.

**Table:** 

Authors' roles & responsibilities	
EHM	Substantial contributions to the conception or design of the work; or the acquisition, analysis, or interpretation of data for the work; drafting the work or revising it critically for important intellectual content; final approval of the version to be published
JCVCG	Substantial contributions to the conception or design of the work; or the acquisition, analysis, or interpretation of data for the work; drafting the work or revising it critically for important intellectual content; final approval of the version to be published
LCA	Substantial contributions to the conception or design of the work; or the acquisition, analysis, or interpretation of data for the work; drafting the work or revising it critically for important intellectual content; final approval of the version to be published
MBW	Substantial contributions to the conception or design of the work; or the acquisition, analysis, or interpretation of data for the work; drafting the work or revising it critically for important intellectual content; final approval of the version to be published
FLC	Substantial contributions to the conception or design of the work; or the acquisition, analysis, or interpretation of data for the work; drafting the work or revising it critically for important intellectual content; final approval of the version to be published
NLB	Substantial contributions to the conception or design of the work; or the acquisition, analysis, or interpretation of data for the work; drafting the work or revising it critically for important intellectual content; final approval of the version to be published;
LCB	Substantial contributions to the conception or design of the work; or the acquisition, analysis, or interpretation of data for the work; drafting the work or revising it critically for important intellectual content; final approval of the version to be published
